# A Novel Non-invasive Approach for Measuring Upper Airway Collapsibility in Mice

**DOI:** 10.3389/fneur.2018.00985

**Published:** 2018-11-20

**Authors:** Yoichi Nishimura, Rafael S. Arias, Huy Pho, Luu Van Pham, Thomaz Fleury Curado, Vsevolod Y. Polotsky, Alan R. Schwartz

**Affiliations:** ^1^Division of Pulmonary and Critical Care Medicine, School of Medicine, Johns Hopkins University, Baltimore, MD, United States; ^2^Department of Otolaryngology, Teikyo University Chiba Medical Center, Chiba, Japan

**Keywords:** obstructive sleep apnea, upper airway collapsibility, critical pressure, pharynx, mice

## Abstract

**Introduction:** Invasive procedures were previously developed for measuring pharyngeal collapsibility in rodents during expiration, when declining neuromuscular activity makes the airway unstable. We developed a non-invasive approach for streamlining collapsibility measurements by characterizing responses in physiologic markers of dynamic expiratory airflow obstruction to negative nasal pressure challenges.

**Methods:** Anesthetized mice were instrumented to monitor upper airway pressure-flow relationships with head-out plethysmography while nasal pressure was ramped down from ~ +5 to −20 cm H_2_O over several breaths. Inspiratory and expiratory flow, volume, and timing characteristics were assessed breath-wise. Pcrit was estimated at transitions in expiratory amplitude and timing parameters, and compared to gold standard P_CRIT_ measurements when nasal and tracheal pressures diverged during expiration. Predictions equations were constructed in a development data set (*n* = 8) and applied prospectively to a validation data set (*n* = 16) to estimate gold standard P_CRIT_.

**Results:** The development data demonstrated that abrupt reversals in expiratory duration and tidal volume during nasal pressure ramps predicted gold standard P_CRIT_ measurements. After applying regression equations from the development to a validation dataset, we found that a combination of expiratory amplitude and timing parameters proved to be robust predictors of gold standard P_CRIT_ with minimal bias and narrow confidence intervals.

**Conclusions:** Markers of expiratory airflow obstruction can be used to model upper airway collapsibility, and can provide sensitive measures of changes in airway collapsibility in rodents. This approach streamlines repeated non-invasive P_CRIT_ measurements, and facilitates studies examining the impact of genetic, environmental, and pharmacologic factors on upper airway control.

## Introduction

Obstructive sleep apnea is a common disorder with an estimated prevalence of 2 to 4% in the general population (1, 2). It is characterized primarily by recurrent occlusion of the upper airway during sleep. Ensuing oxyhemoglobin desaturation and periodic arousals account for the major cardiopulmonary and metabolic morbidity of this disorder (3, 4). Nasal continuous positive airway pressure can treat obstructive sleep apnea by splinting the pharyngeal airway open (5). Although CPAP remains the mainstay of therapy, its acceptance is beset by low adherence to therapy (6). Recently, pharmacologic alternatives to CPAP have been piloted for specific patient subgroups (7–9). Nevertheless, the development of new strategies has been hindered by the lack of easily deployed animal models in which proof of concept studies can be conducted to simulate and treat upper airway obstruction.

Investigators have demonstrated that elevations in pharyngeal collapsibility play a pivotal role in the pathogenesis of obstructive sleep apnea (10–12), as reflected by increases in critical closing pressures (P_CRIT_). Prior studies in large animals have modeled structural alterations and disturbances in upper airway neuromuscular control (13–15), both of which can contribute to elevations in P_CRIT_. More recently, investigators have been able to resolve passive structural and active neuromuscular components of airway collapsibility in rodents (16–18) by characterizing P_CRIT_ from variations in airway pressure-flow dynamics across the respiratory cycle (17–20). In mice, expiratory obstruction develops during ramp decreases in nasal pressure when nasal and tracheal pressures diverge as neuromuscular activity wanes. Investigators have utilized this approach to characterize effects of sleep apnea risk factors (e.g., obesity, central adiposity, and age) (17, 18), neuromuscular activity (21) and neurohumoral factors (e.g., leptin) (18, 22) on airway collapsibility. Nevertheless, monitoring tracheal pressure has posed significant technical challenges and has restricted this approach to non-survival experiments.

The major goal of the current study was to elaborate a streamlined non-invasive method for characterizing airway collapsibility in anesthetized mice during ramp decreases in nasal pressure without monitoring tracheal pressure. We reasoned that negative nasal pressure would obstruct the airway when pharyngeal neuromuscular activity wanes during expiration. Under these circumstances, expiratory obstruction should be characterized by reductions in expiratory airflow, trapped air in the lungs behind the obstruction and a prolongation of expiration. We hypothesized that amplitude and timing indices of expiratory airflow obstruction could be used to estimate gold standard measurements of P_CRIT_, based on tracheal pressure measurements (7, 18). Our findings offer a novel approach to streamlining measurements of P_CRIT_ in both humans and animals.

## Methods

### Approach

In prior work, an approach was developed for measuring P_CRIT_ by lowering the nasal pressure in ramp-like fashion and determining the nasal pressure at which tracheal pressure diverged (17, 18). When nasal pressure fell below a P_CRIT_, we found that nasal pressure was no longer transmitted to the trachea when the airway became most collapsible during the expiratory phase of the respiratory cycle. During inspiration, nasal and tracheal pressures re-equilibrated, indicating that airway patency was restored with phasic upper airway neuromuscular activity. This technique required placement and maintenance of a high-fidelity tracheal pressure signal via tracheostomy during non-survival surgery, which severely limited its applicability on technical grounds and precluded repeated measurements over time.

**Figure 1:**
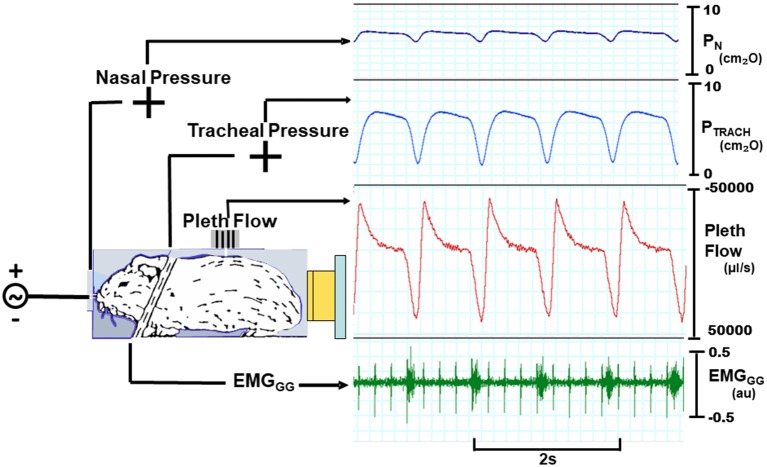
Experimental setup with mouse in head out chamber sealed around neck. Mice were instrumented to monitor upper airway pressure-flow relationships. Sealed snout mask affixed to nostrils with mouth sealed. Nasal and tracheal pressures are monitored, as well as tidal airflow from chamber. A variable pressure source is connected to nose. Inspiratory phasic genioglossus electromyographic activity (EMG_GG_; arbitrary units, au) with cardiac artifact (spikes).

To overcome these limitations, we assessed for signs of expiratory airflow obstruction from the tidal airflow signals during negative nasal pressure ramps (Figure 1). As before, head-out plethysmography was used to monitor tidal airflow continuously (17, 18). This signal was used to derive amplitude and timing indices to determine the nasal pressure at which expiratory airflow obstruction developed (the pharynx collapsed). Specifically, we assessed the impact of progressive decreases in nasal pressure on expiratory peak flow and tidal volume, and on the expiratory duration and duty cycle. P_CRIT_ was estimated by the level of nasal pressure at which the development of expiratory airflow obstruction could be inferred from transitions in these parameters, and compared to gold standard measurements of P_CRIT_. P_CRIT_ estimates were generated from recordings in prior experiments (17, 18).

### Study design

Gold standard and P_CRIT_ estimates were measured in two groups of mice. A development group (*n* = 8) was used to model associations between gold standard P_CRIT_ and predictive physiologic parameters from a minimum of 3 runs in each mouse. Regression equations from the development data set were then applied to data from a validation group (*n* = 16) to examine their accuracy in predicting P_CRIT_. This study was conducted in accordance with the recommendations and approval of the Johns Hopkins Animal Care and Use Committee.

### Mice

Male C57BL/6J (BL6) mice were obtained from Jackson Laboratory (Bar Harbor, ME), housed in a temperature and humidity-controlled micro-isolation facility, fed regular chow and water *ad libitum*, and studied at ~14 weeks of age (Table 1). The study protocols were approved by the Johns Hopkins Animal Care and Use Committee (JHACUC), and all animal experiments were conducted in accordance with the JHACUC guidelines. Age and weight are reported for male mice from studies in previous experiments on mouse upper airway control (17, 18).

**Table 1:** C57BL/6J Mouse characteristics.

	**n**	**Age (week)**		**Weight (g)**	
		**Mean ± SE**	**Range**	**Mean ± SE**	**Range**
Development data set	8	13.6 ± 1.6	9.0–19.0	26.6 ± 1.4	23.2–34.4
Validation data set	16	14.7 ± 1.9	9.0–29.0	29.1 ± 1.0	24.2–34.7

### Experimental setup, procedures and protocol

Mice were instrumented and studied experimentally, as previously described (17, 18). In brief, isoflurane anesthesia was titrated between 0.5 and 1.5% (usually ~1%) to target a respiratory rate in the range of 60 to 80 per min. Atropine was injected (0.001 mg I.P.) to minimize airway secretions, and body temperature was maintained at 36.5–37.5°C. The trachea was then cannulated with a tapered cannula through a midline incision and the cannula was secured with sutures. Two Teflon coated fine wires were also tunneled subcutaneously and sutured to the ventral surface of the geniohyoid/genioglossus muscle group bilaterally. The mouth was then sealed shut. The mouse was then placed into a head-out plethysmograph in the prone position (17, 18).

The experimental setup for making pressure-flow recordings consisted of the following. A low-dead space, tight fitting nasal cannula was placed over the snout, and connected to a blow-by breathing circuit through which fresh oxygen and isoflurane were administered. The nasal pressure (P_N_) and tracheal pressure (P_TRACH_) were monitored with differential pressure transducers referenced to atmospheric pressure. A calibrated laminar flow pneumotachometer was mounted onto the plethysmograph and connected to a differential pressure transducer. All pressure and airflow signals were amplified and digitized for real-time display, storage and data analysis. The genioglossus electromyographic activity (EMG_GG_) signal was amplified, band-pass-filtered from 30 to 1,000 Hz, digitized at 1 KHz, rectified and the moving average was computed with a 55 ms time constant.

**Figure 2:**
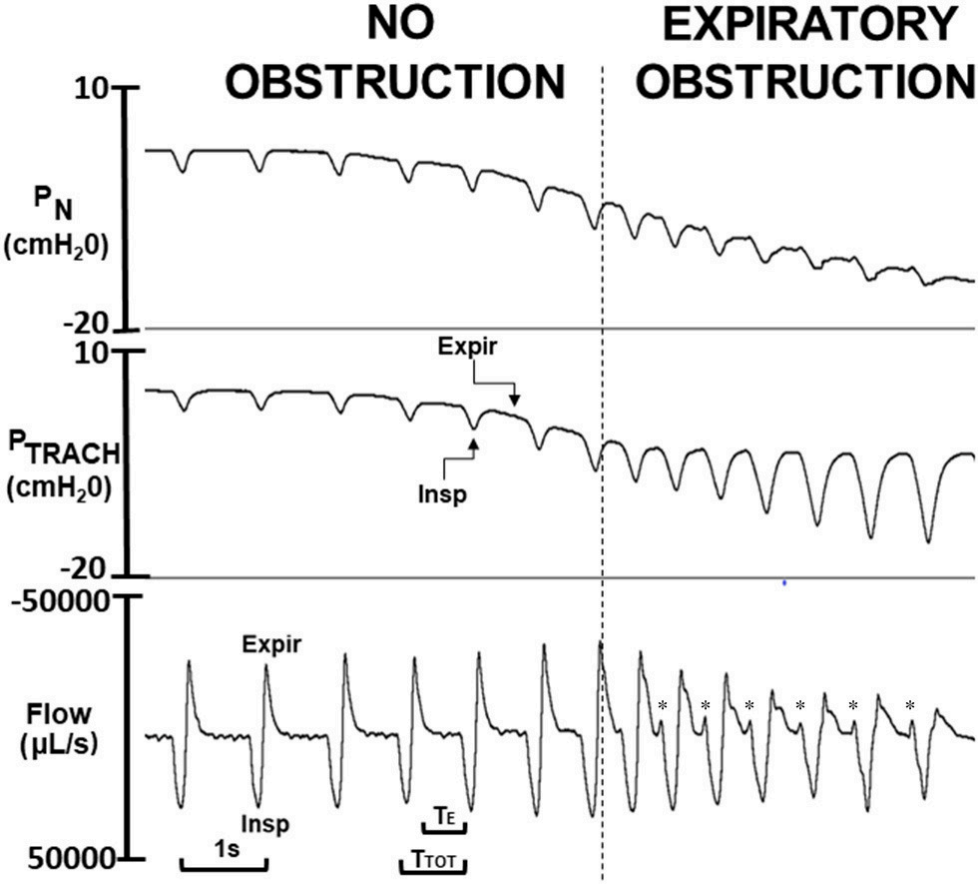
Critical pressure (P_CRIT_) determined from ramp decrease in nasal pressure (P_N_) over a series of breaths from approximately +5 to −20 cm H_2_O. As P_N_ decreased progressively, expiratory P_N_ and tracheal pressure (P_TRACH_) tracked one another over the initial series of breaths (to left of vertical dashed line), indicating airway patency. Once nasal pressure fell below P_CRIT_ (to right of vertical line), nasal and tracheal pressures diverged during expiration (Expir) but not during inspiration (Insp), indicating expiratory obstruction. Physiologic markers for expiratory airflow obstruction were observed in tidal airflow response to decreasing P_N_ including reversals in expiratory peak flow, tidal volume (area under expiratory vs. inspiratory flow trace), and time (T_E_) and duty cycle (T_E_/T_TOT_). At end-expiration, a sudden jet of expiratory flow (see ^*^) coincides with onset of phasic genioglossus electromyographic activity (not shown), indicating release of trapped air when the pharynx reopens.

To determine P_CRIT_, P_N_ was lowered in ramp-like fashion from ~ +5 cmH_2_O to ~ −20 cmH_2_O (Figure 2). Before each P_N_ ramp, the patency of the tracheostomy tube was assured by flushing the tracheal cannula. At the end of the study, mice were euthanized with an overdose of pentobarbital (60 mg IP).

### Data analysis

Upper airway function was assessed during expiration (17, 18), when EMG_GG_ fell to tonic levels. Gold standard measurements of passive P_CRIT_ were defined by the P_N_ at which end-expiratory P_TRACH_ diverged with further decreases in P_N_. We previously demonstrated that site of expiratory obstruction in this mouse model was located rostral to the palatal rim, and was indistinguishable from P_CRIT_ measurements during complete neuromuscular blockade (17).

**Figure 3:**
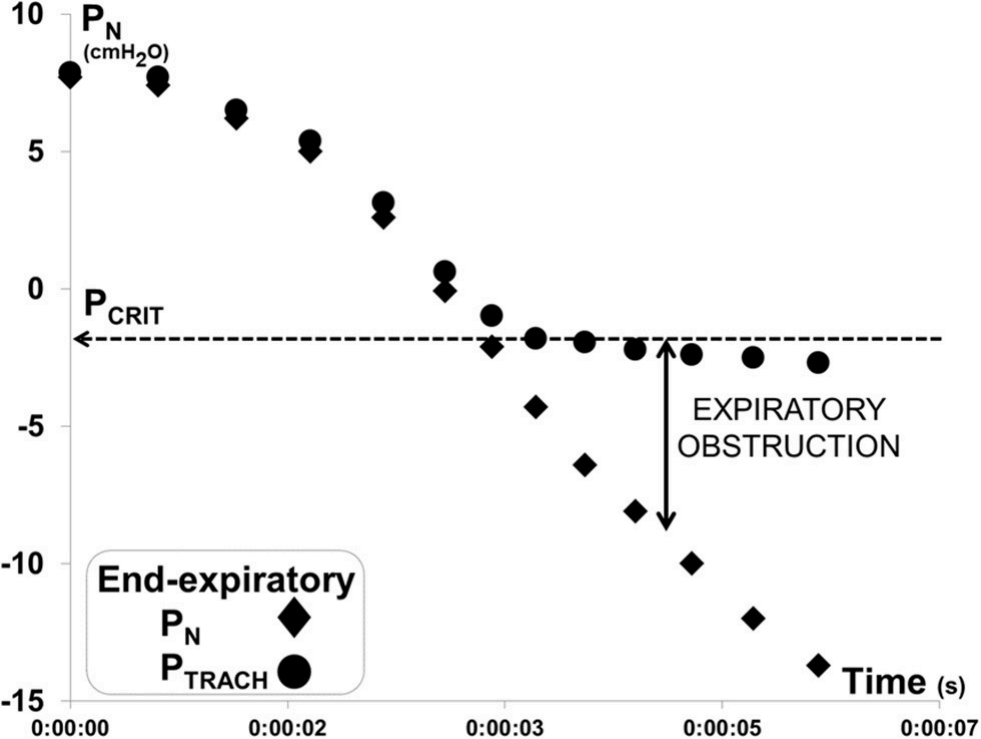
Method for measuring gold standard critical pressure (P_CRIT_). Expiratory pressure gradient developed between nasal (P_N_) and tracheal (P_TRACH_) pressures during a ramp decrease in P_N_ for the recording illustrated in Figure 2. P_N_ and P_TRACH_ diverged, indicating that the airway obstructed when P_N_ fell below a critical pressure (P_CRIT_ at horizontal dashed line). Data points represent P_N_ (diamonds) and P_TRACH_ (circles) measurements from each breath in run.

**Figure 4:**
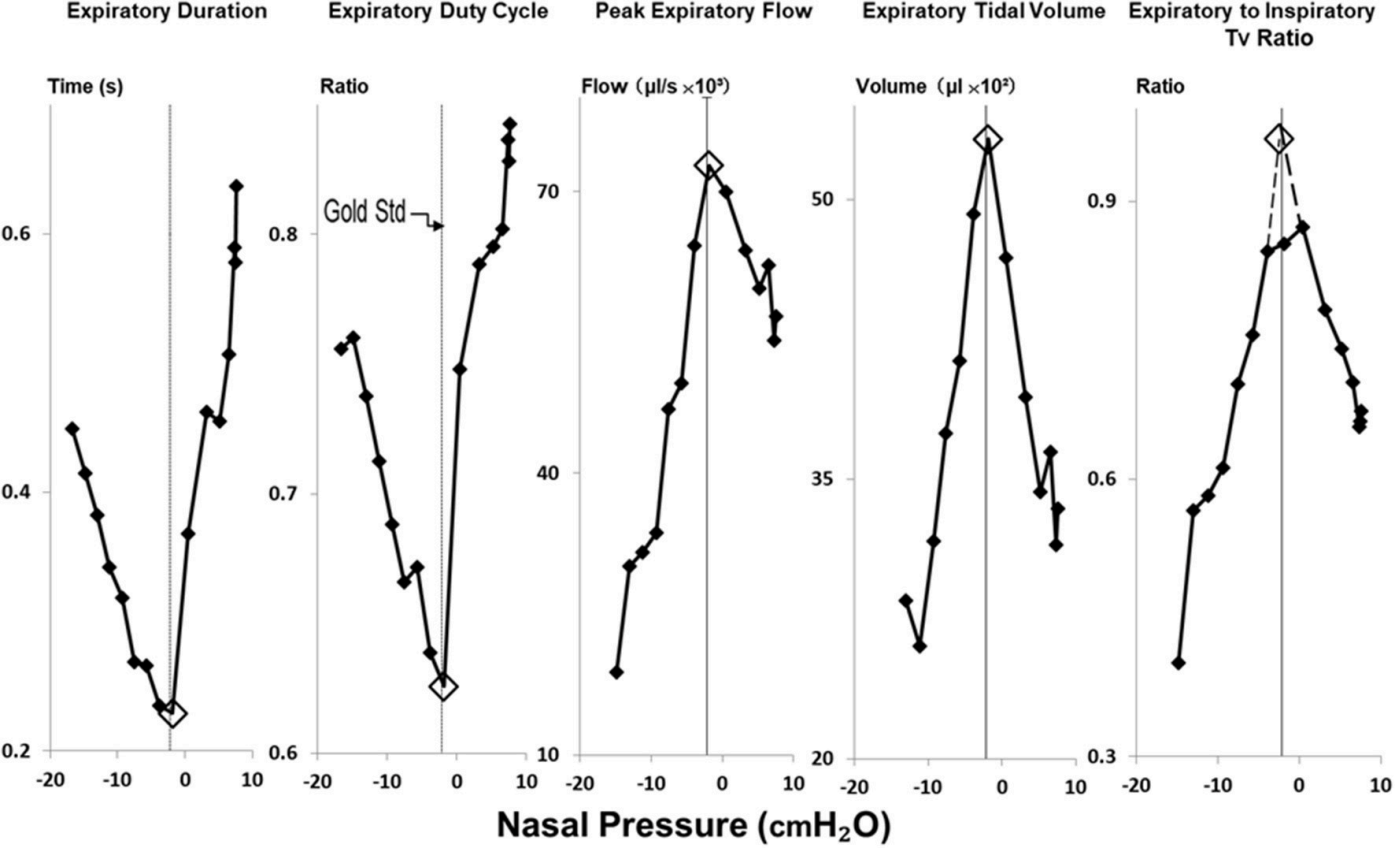
Method for estimating gold standard P_CRIT_ with physiologic surrogates for expiratory obstruction. Reversals in expiratory duration, expiratory duty cycle, peak expiratory flow, expiratory tidal volume and ratio of expiratory to inspiratory tidal volumes (Tv) occurred at the same breath in negative pressure ramp illustrated in Figure 2. Open diamonds highlight the breath at which these transitions occurred, and the corresponding nasal pressure used to estimate the critical pressure. P_CRIT_ estimates and gold standard measurements (at thin vertical line in each graph) coincide well.

Each P_N_ ramp (run) was evaluated to determine P_CRIT_ as the P_N_ at which further decreases in P_N_ were no longer transmitted to P_TRACH_ at end-expiration, as previously described (17, 18) (Figure 3). To ensure precision in P_CRIT_ measurements, a minimum of 5 breaths were required to assess P_CRIT_ within a range of P_N_ from 0 to −10 cmH_2_O, thereby providing sufficient resolution to discern a divergence in P_N_ and P_TRACH_. Accurate estimates of P_CRIT_ were also ensured by imposing a requirement for an abrupt change or reversal in amplitude and timing parameters over the course of the nasal pressure ramp, as shown in Figure 4.

Physiologic markers of expiratory obstruction were derived from the tidal airflow signal and plotted against the mean end-expiratory nasal pressure at the start and end of each breath during ramps as follows. *Peak expiratory airflow* was measured directly from the expiratory flow waveform. Inspiratory and expiratory tidal volumes were calculated by integrating airflow signals during each phase of the respiratory cycle. This signal was used to derive the *expiratory tidal volume* and the *ratio of expiratory to inspiratory tidal volume* (referred to subsequently as the *tidal volume ratio*). Respiratory timing indices were also measured including the *expiratory duration* (T_E_) and the *expiratory duty cycle* (T_E_/T_TOT_) where T_TOT_ is the period of the entire respiratory cycle. As can be seen in Figure 2, the onset of expiratory flow obstruction was also associated with an increased expiratory time constant and a pre-inspiratory jet of expiratory airflow. The latter coincided with the onset of phasic inspiratory activity, and represented the release of trapped air from the lungs when the airway reopened (see below).

Amplitude and timing parameters were then plotted against P_N_. These plots were used to estimate P_CRIT_ as the level of P_N_ at which breath-wise transitions or reversals in these parameters occurred (Figure 4), consistent with the development of expiratory airflow obstruction. P_CRIT_ values were estimated for each index of expiratory airway obstruction and compared to gold standard P_CRIT_ measurements.

### Statistical analysis

Our analytic plan was designed (1) to compare physiologic surrogates for expiratory airflow obstruction with gold standard P_CRIT_ measurements in a development data set, and to (2) predict P_CRIT_ in a validation sample. In the development data set, linear regression was used to model the relationship between gold standard and each P_CRIT_ estimate. These predictive equations were then applied to the validation data set to determine how well each P_CRIT_ estimate predicted the gold standard P_CRIT_. Bland-Altman analysis was applied to compare differences between physiologic markers and gold standard P_CRIT_, and to determine the bias and confidence intervals for each physiologic surrogate with gold standard P_CRIT_. In each data set, mean values for gold standard and P_CRIT_ estimates were used to model these relationships. All analyses were conducted in XLSTAT (Microsoft Inc.). Statistical significance was inferred at a *p* < 0.05 level. Values were expressed as means ± SE.

## Results

### Illustrative nasal pressure ramp

In Figure 4, amplitude and timing parameters are illustrated for each breath in the nasal pressure ramp shown in Figure 2. P_CRIT_ estimates are illustrated for each parameter at the point that it reverses course (see transition breath at open diamonds, Figure 4), and coincide with the gold standard P_CRIT_ (see vertical thin lines). In each graph, an abrupt change in slope occurs between non-obstructed breaths at the start of the ramp and obstructed breaths at the end of the ramp. Initial decreases in expiratory duration and duty cycle, and increases in peak expiratory flow, tidal volume and the expiratory to inspiratory tidal volume ratio can be seen as nasal pressure is lowered progressively, consistent with an increased driving pressure to expiratory flow. Further decreases in nasal pressure beyond the gold standard P_CRIT_, however, result in progressive increases in expiratory duration and duty cycle and reductions in expiratory peak flow, tidal volume, and tidal volume ratio. A sudden reversal in each parameter can be attributed to the development of expiratory obstruction, as indicated by a divergence in expiratory nasal and tracheal pressure at the gold standard P_CRIT_ (Figures 2, 3).

### Development data set

In a limited development data set, linear regression models demonstrated that the nasal pressure at which the expiratory duration started to lengthen during nasal pressure ramps was a significant predictor of the gold standard P_CRIT_ and accounted for 54% of the variance of this parameter (Table 2, *p* = 0.038). Similarly, a trend was also detected between the nasal pressure at which expiratory tidal volume began to fall and the gold standard P_CRIT_ (Table 2, *p* = 0.121). Reversals in other amplitude and timing parameters, however, were not associated with gold standard P_CRIT_ measurements (Table 2, *p* = n.s.).

**Table 2:** Regression models for gold standard vs. estimated P_CRIT_ from timing and amplitude criteria in development data set (*n* = 8).

**Parameter**	**β**	**Constant**	**R-square**	***p-*value**
**TIMING**				
Expiratory duration (T_E_)	1.68	2.21	0.54	0.038
Expiratory duty cycle (EDC)	1.19	0.82	0.19	0.288
**AMPLITUDE**				
Peak expiratory flow (P_K_)	0.34	−3.05	0.07	0.556
Expiratory tidal volume(T_V_)	0.64	−1.56	0.35	0.121
Expiratory TV/inspiratory T_V_(T_V_ ratio)	−0.26	−3.70	0.03	0.711

### Validation data set

**Figure 5:**
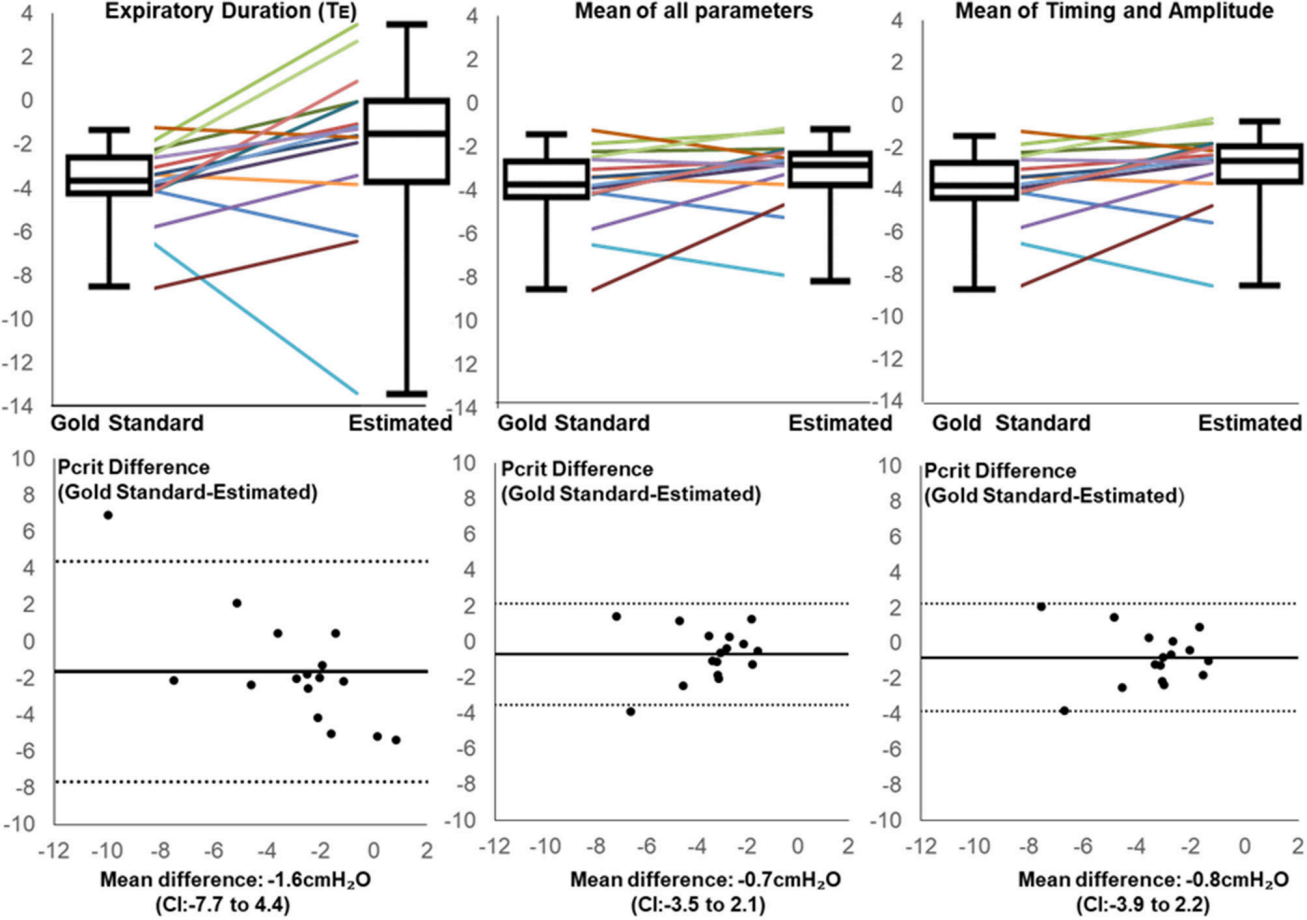
Line and box plots for Estimated and Gold Standard P_CRIT_ in validation group based on Expiratory Duration alone (upper left panel), mean values for all physiologic surrogates (upper middle panel) and mean of timing and amplitude mean surrogates (upper right panel), along with corresponding Bland-Altman plots (lower panels). Bias and confidence intervals (CI) decrease when surrogate means are used to estimate P_CRIT_ (middle and right vs. left lower panels).

After applying regression equations generated from the development to the validation data set, we found that the association between the transition in expiratory duration and P_CRIT_ persisted (*p* = 0.004, Table 3). We also found that associations remained significant when the mean of all parameters and when the means of timing and amplitude means were used to predict the gold standard P_CRIT_ (Figure 5, upper panels). Bland-Altman plots for each set of predictor variables demonstrated little bias in estimates of P_CRIT_ (Figure 5, lower panels). Nevertheless, substantial reductions in the overall bias and confidence intervals (CI) around P_CRIT_ estimates occurred based on the mean of all parameters and on the mean of the means for timing and amplitude parameters (Figure 5, lower panels). The latter indicates that mean expiratory timing and amplitude parameters provide robust estimates of P_CRIT_ in anesthetized mice.

**Table 3:** Regression models for gold standard vs. estimated P_CRIT_ from timing and amplitude criteria in validation data set (*n* = 16).

**Parameter(s)**	**β**	**Constant**	**R-square**	***p-*value**
Expiratory duration (T_E_) alone	0.34	−3.14	0.47	0.004
Mean of all parameters^*^	0.74	−1.50	0.44	0.005
Mean of timing and amplitude^†^	0.64	−1.91	0.45	0.005

* *Mean of all 5 parameters equally weighted*.

† *Mean of timing and amplitude parameter means*.

## Discussion

Two salient findings emerged from the present study. First, our development data set demonstrate a consistent relationship between gold standard measurements of P_CRIT_ and specific markers of expiratory airflow obstruction, viz., expiratory duration and the expiratory tidal volume. Second, after applying regression equations from the development to a validation data set, we found that a combination of expiratory amplitude and timing parameters proved to be robust predictors of gold standard P_CRIT_ with minimal bias and narrow confidence intervals. Taken together, our findings suggest that markers of expiratory airflow obstruction can be used to model upper airway collapsibility under passive (hypotonic) conditions, and that these markers can provide sensitive measures of changes in airway collapsibility in rodent models of obstructive sleep apnea. This approach obviates the need for placing a tracheal cannula (as required for making gold standard P_CRIT_ measurements), and can thereby facilitate non-invasive repeated P_CRIT_ measurements in rodents over time.

**Figure 6:**
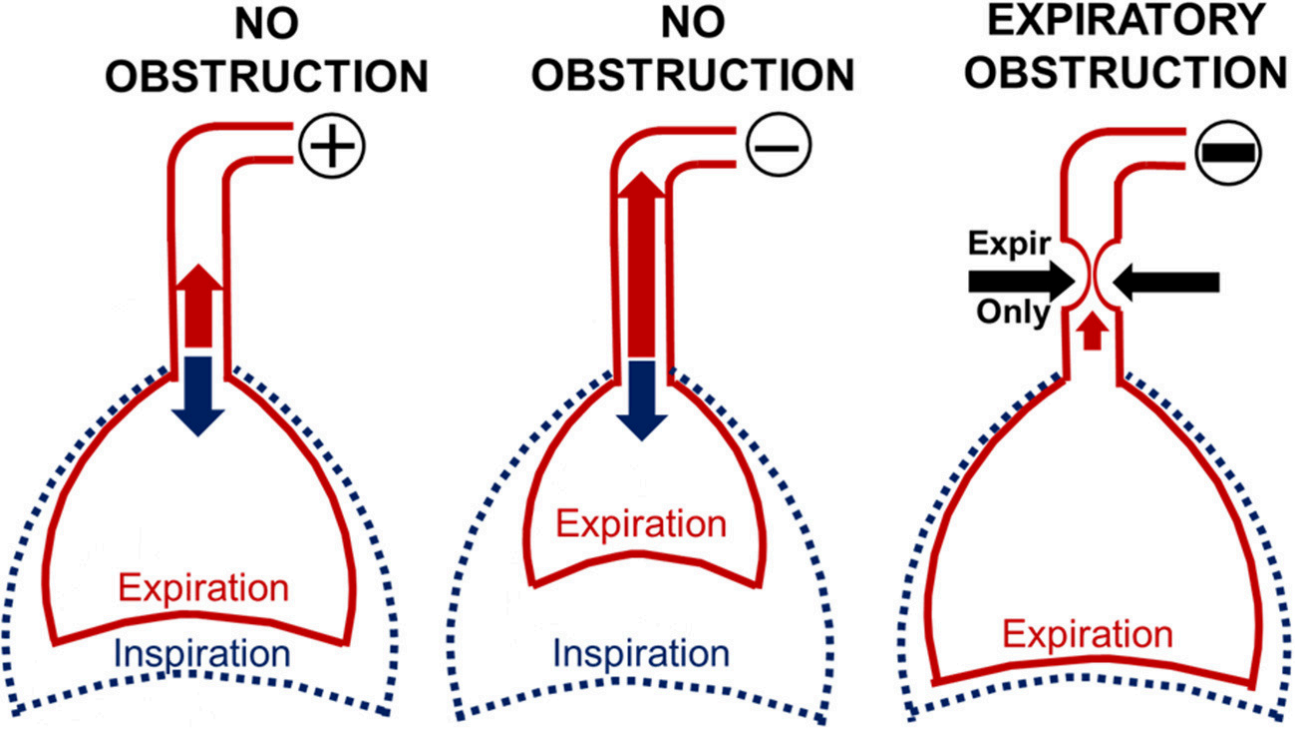
Modeling the effect of expiratory obstruction on respiratory timing and amplitude indices. When nasal pressure (P_N_) was 0 or positive, inspiration and expiration remained unimpeded (L panel). Initial reductions in P_N_ pulled air from the lungs and augmented expiration (middle panel) while inspiration remained unchanged (middle panel). In contrast, further reductions in nasal pressure collapsed the pharynx during expiration (R panel), leading to decreases in expiratory peak flow and tidal volume. Expiratory air trapping activated pulmonary stretch receptors (Herring Breuer reflex), which prolonged expiratory duration and duty cycle. Phasic activity in pharyngeal muscles, however, restored airway patency, allowing inspiration to proceed normally.

Our findings demonstrate marked changes in expiratory pressure-flow dynamics during negative pressure ramps between periods with and without upper airway obstruction. These differences are related to dynamic increases in airway collapsibility during expiration when pharyngeal neuromuscular activity wanes in anesthetized rodents (16–18). During expiration, pharyngeal collapsibility is largely determined by the mechanical properties of the upper airway, which rises to levels observed during complete neuromuscular blockade (17, 23). At first, progressive reductions in nasal pressure led to prompt increases in expiratory flow and tidal volumes (Figure 6, L vs. middle panel). As long as the airway remained fully patent during inspiration, inspiratory flow and tidal volume were unaffected (Figure 6, middle panel). As nasal pressure decreased further, the airway collapsed when pharyngeal neuromuscular activity waned during expiration. Expiratory flow obstruction (Figure 6, R panel) resulted in a decrease in peak expiratory flow and tidal volume with trapped air inside the lung. Increases in end-expiratory lung volume activated pulmonary stretch receptors (Herring-Breuer reflex) (15, 24), which accounts for observed increases in expiratory duration and expiratory duty cycle. In contrast, inspiratory tidal volumes remained well-preserved, since phasic neuromuscular activity restored airway patency during this phase in the respiratory cycle (13, 14). Thus, abrupt reversals in expiratory duration and duty cycle, and in peak expiratory flow and tidal volume signified the development of dynamic expiratory airway collapse during nasal pressure ramps.

Our findings on expiratory flow dynamics during ramp decreases in nasal pressure have both practical as well as theoretical implications. In prior studies, the placement and maintenance of a tracheal cannula required significant training, skill and attention to signal quality. The current approach obviates the need for invasive tracheal pressure monitoring and simplifies the experimental preparation. Identifying physiologic markers of expiratory airflow obstruction allows us to streamline methods for estimating passive P_CRIT_ in anesthetized mice. In so doing, the current approach will make this method accessible to investigators seeking to increase throughput in studies examining the impact of genetic, environmental and pharmacologic factors on upper airway collapsibility. Eliminating the need for tracheostomy and tracheal intubation will also facilitate repeated P_CRIT_ within-mouse assessments in response to interventions. This non-invasive approach allows mice to survive experimental treatments and other complementary measurements (e.g., imaging).

Several limitations should be considered in evaluating our study findings. First, all measurements were made in a stable state of anesthesia, which is known to slow the respiratory rate, augment phasic inspiratory neuromuscular activity and decrease tonic expiratory activity. Phasic differences in the control of airway mechanics predispose to expiratory obstruction and marked transitions in expiratory amplitude and timing indices during negative nasal pressure ramps. Second, inflection points in these indices did not all occur uniformly with the onset of expiratory obstruction. Nevertheless, a predominant pattern emerged with respect to several markers of expiratory obstruction, which mutually reinforced the conclusion that collapse had occurred at specific levels of nasal pressure. Third, although the precise anatomic correlates of pharyngeal collapse have not been delineated, we have previously isolated the obstruction to the velopharyngeal airway (17), most likely due to collapse at the palatal rim (14, 25). Fourth, we recognize that our method was specifically designed to assess pharyngeal collapsibility during expiration (when EMG activity was relatively low), reflecting structural rather than neuromuscular determinants of airway collapsibility (16). We therefore targeted expiratory P_CRIT_ measurements by adjusting isoflurane concentration to lower the respiratory rate and prolong expiration. Under these circumstances, we have previously shown that our expiratory Pcrit measurements are indistinguishable from those during complete neuromuscular blockade (17). Fifth, we acknowledge that our findings may not be generalizable to other anesthetic agents or to other strains of mice across the age and weight range.

Our findings of expiratory obstruction in mice have broad applicability to studies in both rodents and humans. Reductions in pharyngeal neuromuscular activity during expiration can predispose both species to expiratory airflow obstruction. A unique advantage of our methodology is that it permits a dynamic assessment of airway collapsibility under passive conditions when airway neuromuscular activity is quiescent. As such, the approach may be ideally suited to evaluate the impact of anatomic structures on airway collapsibility, particularly in assessing patients during drug-induced sleep endoscopy for specific types of airway reconstructive (26–28) or hypoglossal stimulation surgery (29). Our methodology also builds the foundation for examining the impact of pharmacologic agents and chemogenetics to treating obstructive sleep apnea in proof-of-concept murine studies (30, 31). Finally, streamlining the physiologic assessment of upper airway collapsibility can serve to elucidate functional effects of genetic defects in inbred murine strains (32, 33). Our approach holds the potential for streamlining measurements of P_CRIT_ and facilitating research across species and interventions.

## Author contributions

YN, AS collected and analyzed the data, conducted statistical analysis, and prepared the manuscript. RA collected and analyzed the data. HP, LP, and TC analyzed the data and conducted statistical analysis. VP prepared the manuscript.

### Conflict of interest statement

The authors declare that the research was conducted in the absence of any commercial or financial relationships that could be construed as a potential conflict of interest.

## References

[1] Peppard PE Young T Barnet JH Palta M Hagen EW Hla KM. Increased prevalence of sleep-disordered breathing in adults. Am J Epidemiol. (2013) 177:1006–14. 10.1093/aje/kws342 23589584 PMC3639722

[2] Young T Peppard P Palta M Hla KM Finn L Morgan B. Population-based study of sleep-disordered breathing as a risk factor for hypertension. Arch Intern Med. (1997) 157:1746–52. 10.1001/archinte.1997.00440360178019 9250236

[3] Punjabi NM. The epidemiology of adult obstructive sleep apnea. Proc Am Thorac Soc. (2008) 5:136–43. 10.1513/pats.200709-155MG 18250205 PMC2645248

[4] Young T Peppard PE Gottlieb DJ. Epidemiology of obstructive sleep apnea: a population health perspective. Am J Respir Crit Care Med. (2002) 165:1217–39. 10.1164/rccm.2109080 11991871

[5] Sullivan CE Issa FG Berthon-Jones M Eves L. Reversal of obstructive sleep apnoea by continuous positive airway pressure applied through the nares. Lancet (1981) 1:862–5. 10.1016/S0140-6736(81)92140-1 6112294

[6] Weaver TE Grunstein RR. Adherence to continuous positive airway pressure therapy: the challenge to effective treatment. Proc Am Thorac Soc. (2008) 5:173–8. 10.1513/pats.200708-119MG 18250209 PMC2645251

[7] Eckert DJ Owens RL Kehlmann GB Wellman A Rahangdale S Yim-Yeh S. Eszopiclone increases the respiratory arousal threshold and lowers the apnoea/hypopnoea index in obstructive sleep apnoea patients with a low arousal threshold. Clin Sci. (2011) 120:505–14. 10.1042/CS20100588 21269278 PMC3415379

[8] Edwards BA Connolly JG Campana LM Sands SA Trinder JA White DP. Acetazolamide attenuates the ventilatory response to arousal in patients with obstructive sleep apnea. Sleep (2013) 36:281–5. 10.5665/sleep.2390 23372276 PMC3543060

[9] Edwards BA Sands SA Owens RL Eckert DJ Landry S White DP. The Combination of supplemental oxygen and a hypnotic markedly improves obstructive sleep apnea in patients with a mild to moderate upper airway collapsibility. Sleep (2016) 39:1973–83. 10.5665/sleep.6226 27634790 PMC5070751

[10] Eckert DJ White DP Jordan AS Malhotra A Wellman A. Defining phenotypic causes of obstructive sleep apnea. Identification of novel therapeutic targets. Am J Respir Crit Care Med. (2013) 188:996–1004. 10.1164/rccm.201303-0448OC 23721582 PMC3826282

[11] Gold AR Schwartz AR. The pharyngeal critical pressure. The whys and hows of using nasal continuous positive airway pressure diagnostically. Chest (1996) 110:1077–88. 10.1378/chest.110.4.1077 8874271

[12] Schwartz AR O'Donnell CP Baron J Schubert N Alam D Samadi SD. The hypotonic upper airway in obstructive sleep apnea: role of structures and neuromuscular activity. Am J Respir Crit Care Med. (1998) 157:1051–7. 9563718 10.1164/ajrccm.157.4.9706067

[13] Schwartz AR Thut DC Russ B Seelagy M Yuan X Brower RG. Effect of electrical stimulation of the hypoglossal nerve on airflow mechanics in the isolated upper airway. Am Rev Respir Dis. (1993) 147:1144–50. 10.1164/ajrccm/147.5.1144 8484623

[14] Schwartz AR Thut DC Brower RG Gauda EB Roach D Permutt S. Modulation of maximal inspiratory airflow by neuromuscular activity: effect of CO2. J Appl Physiol. (1993) 74:1597–605. 10.1152/jappl.1993.74.4.1597 8514673

[15] Seelagy MM Schwartz AR Russ DB King ED Wise RA Smith PL. Reflex modulation of airflow dynamics through the upper airway. J Appl Physiol. (1994) 76:2692–700. 10.1152/jappl.1994.76.6.2692 7928902

[16] Liu A Pichard L Schneider H Patil SP Smith PL Polotsky V. Neuromechanical control of the isolated upper airway of mice. J Appl Physiol. (2008) 105:1237–45. 10.1152/japplphysiol.90461.2008 18653751 PMC2576036

[17] Polotsky M Elsayed-Ahmed AS Pichard LE Richardson RA Smith PL Schneider H. Effect of age and weight on upper airway function in a mouse model. J Appl Physiol. (2011) 111:696–703. 10.1152/japplphysiol.00123.2011 21719728 PMC3174798

[18] Polotsky M Elsayed-Ahmed AS Pichard L Harris CC Smith PL Schneider H. Effects of leptin and obesity on the upper airway function. J Appl Physiol. (2012) 112:1637–43. 10.1152/japplphysiol.01222.2011 22345430 PMC3365404

[19] Kirkness JP Schwartz AR Patil SP Pichard LE Marx JJ Smith PL. Dynamic modulation of upper airway function during sleep–a novel single breath method. J Appl Physiol. (2006) 101:1489–94. 10.1152/japplphysiol.00173.2006 16825526

[20] Schneider H Boudewyns A Smith PL O'Donnell CP Canisius S Stammnitz A. Modulation of upper airway collapsibility during sleep: influence of respiratory phase and flow regimen. J Appl Physiol. (2002) 93:1365–76. 10.1152/japplphysiol.00942.2001 12235037

[21] Fuller D Williams JS Janssen PL Fregosi RF. Effect of co-activation of tongue protrudor and retractor muscles on tongue movements and pharyngeal airflow mechanics in the rat. (1999) J. Physiol. 519(Pt 2):601–13. 10.1111/j.1469-7793.1999.0601m.x 10457075 PMC2269504

[22] Nakano H Magalang UJ Lee SD Krasney JA Farkas GA. Serotonergic modulation of ventilation and upper airway stability in obese Zucker rats. Am J Respir Crit Care Med. (2001) 163:1191–7. 10.1164/ajrccm.163.5.2004230 11316658

[23] Brouillette RT Thach BT. A neuromuscular mechanism maintaining extrathoracic airway patency. J Appl Physiol. (1979) 46:772–9. 10.1152/jappl.1979.46.4.772 457556

[24] Van Lunteren E Strohl KP Parker DM Bruce EN Van de Graaff WB Cherniack NS Phasic volume-related feedback on upper airway muscle activity. J. Appl. Physiol. (1984) 56:730–6. 10.1152/jappl.1984.56.3.730 6706778

[25] Thut DC Schwartz AR Roach D Wise RA Permutt S Smith PL. Tracheal and neck position influence upper airway airflow dynamics by altering airway length. J Appl Physiol. (1993) 75:2084–90. 10.1152/jappl.1993.75.5.2084 8307863

[26] Blumen M Bequignon E Chabolle F. Drug-induced sleep endoscopy: a new gold standard for evaluating OSAS? Part I: technique. Eur Ann Otorhinolaryngol Head Neck Dis. (2017) 134:101–7. 10.1016/j.anorl.2016.11.005 28279631

[27] Blumen M Bequignon E Chabolle F. Drug-induced sleep endoscopy: a new gold standard for evaluating OSAS? Part II: results. Eur Ann Otorhinolaryngol Head Neck Dis. (2017) 134:109–15. 10.1016/j.anorl.2016.12.004 28279632

[28] Kezirian EJ Hohenhorst W de Vries N. Drug-induced sleep endoscopy: the VOTE classification. Eur Arch Otorhinolaryngol. (2011) 268:1233–6. 10.1007/s00405-011-1633-8 21614467

[29] Strollo PJ Jr Soose RJ Maurer JT de VN Cornelius J Froymovich O. Upper-airway stimulation for obstructive sleep apnea. N Engl J Med. (2014) 370:139–49. 10.1056/NEJMoa1308659 24401051

[30] Curado TF Fishbein K Pho H Brennick M Dergacheva O Sennes LU Chemogenetic stimulation of the hypoglossal neurons improves upper airway patency. Sci Rep. (2017) 7:44392 10.1038/srep44392 28281681 PMC5345079

[31] Horton GA Fraigne JJ Torontali ZA Snow MB Lapierre JL Liu H. Activation of the hypoglossal to tongue musculature motor pathway by remote control. Sci Rep. (2017) 7:45860. 10.1038/srep45860 28383527 PMC5382915

[32] Brennick MJ Pack AI Ko K Kim E Pickup S Maislin G. Altered upper airway and soft tissue structures in the New Zealand Obese mouse. Am J Respir Crit Care Med. (2009) 179:158–69. 10.1164/rccm.200809-1435OC 18996996 PMC2633061

[33] Brennick MJ Kuna ST Pickup S Cater J Schwab RJ. Respiratory modulation of the pharyngeal airway in lean and obese mice. Respir Physiol Neurobiol. (2011) 175:296–302. 10.1016/j.resp.2010.12.006 21167963 PMC3032032

